# Alcohol Consumption and Its Influence on the Clinical Picture of Puumala Hantavirus Infection

**DOI:** 10.3390/v14030500

**Published:** 2022-02-28

**Authors:** Laura Tervo, Tuula K. Outinen, Satu Mäkelä, Jenna Mustalahti, Heini Huhtala, Ilkka Pörsti, Jaana Syrjänen, Jukka T. Mustonen, Onni Niemelä

**Affiliations:** 1 Department of Internal Medicine, Tampere University Hospital, 33520 Tampere, Finland; tuula.outinen@gmail.com (T.K.O.); satu.m.makela@pshp.fi (S.M.); jenna.h.mustalahti@gmail.com (J.M.); ilkka.porsti@tuni.fi (I.P.); jaana.syrjanen@pshp.fi (J.S.); jukka.mustonen@tuni.fi (J.T.M.); 2 Faculty of Medicine and Health Technology, Tampere University, 33520 Tampere, Finland; onni.niemela@epshp.fi; 3 Faculty of Social Sciences, Tampere University, 33520 Tampere, Finland; heini.huhtala@tuni.fi; 4 Department of Laboratory Medicine and Medical Research Unit, Seinäjoki Central Hospital, 60220 Seinäjoki, Finland

**Keywords:** Puumala hantavirus, hemorrhagic fever with renal syndrome, alcohol drinking, ethanol, liver enzymes, pancreatitis, acute kidney injury

## Abstract

Puumala hantavirus (PUUV) causes hemorrhagic fever with renal syndrome. Characteristic clinical findings include acute kidney injury (AKI), thrombocytopenia, and capillary leakage. Smoking increases the risk of severe AKI, but it is not known whether alcohol consumption predisposes patients to a more severe infection. Liver and pancreatic enzymes, as well as biomarkers of alcohol consumption (gamma-glutamyl transferase, GGT; carbohydrate-deficient transferrin, CDT; GGT-CDT combination; and ethyl glucuronide, EtG), were measured from 66 patients with acute PUUV infection during hospitalization and at the convalescence phase. Alcohol consumption was present in 41% of the study population, 15% showing signs of heavy drinking. Alcohol use did not affect the severity of PUUV induced AKI nor the overall clinical picture of the infection. Liver enzyme levels (GGT or alanine aminotransferase, ALT) were elevated in 64% of the patients, but the levels did not associate with the markers reflecting the severity of the disease. Serum amylase activities at the convalescent stage were higher than those at the acute phase (*p* < 0.001). No cases with acute pancreatitis were found. In conclusion, our findings indicate that alcohol consumption does not seem to affect the clinical course of an acute PUUV infection.

## 1. Introduction

Nephropathia epidemica (NE) is a mild form of hemorrhagic fever with renal syndrome (HFRS). It is caused by Puumala hantavirus (PUUV), found in Europe and Russia [1]. The PUUV infection is carried by the bank vole (*Myodes glareolus*), and the infection is transmitted to humans by inhaling the aerosols of infected rodent excreta [2]. Other hantaviruses causing HFRS are Hantaan, Dobrava, and Seoul viruses [2]. The fatality rate of NE is low, ranging from 0.08% up to 0.4% [3,4]. The disease burden in Finland is considerable with the incidence of diagnosed PUUV infections of 31 to 39 cases per 100,000 inhabitants [5]. According to a recent nationwide study, a seroprevalence of 12.5% in the Finnish population was found [6].

The clinical picture of PUUV infection varies. Typical symptoms are fever, headache, nausea, abdominal pain, and backache [2,7]. The most typical laboratory findings are low platelet count, leukocytosis, anemia, and elevated plasma C-reactive protein (CRP) and creatinine levels, as well as hematuria and proteinuria [8]. A PUUV infection can be divided into five distinct phases: febrile, hypotensive, oliguric, polyuric, and convalescent phases [8]. Renal involvement includes the oliguric phase with acute kidney injury (AKI), followed by polyuria and spontaneous recovery. A small percentage of patients need transient hemodialysis treatment [9]. Host genes have an influence on the outcome of PUUV infection [10,11,12]. Several biomarkers can predict the severity of the disease [8,13,14]. Recently, albuminuria, hematuria, and glucosuria, as well as hyperglycemia have been shown to predict the severity of PUUV infection [15,16,17].

Raised serum aminotransferases (ALT, AST) are reported as typical laboratory findings in HFRS, with a prevalence of 41–85% [18,19,20,21]. In a Swedish study, 60% of patients exceeded the upper normal limits [19]. The association of aminotransferase levels with the clinical course or symptoms in PUUV infection has, however, remained unknown.

The results concerning pancreatic involvement in HFRS have been contradictory in previous studies. There are reports of a small series of HFRS accompanied by acute pancreatitis [22,23,24,25,26,27,28]. In these studies, different hantaviruses are presented. To our knowledge, there is only one case report of PUUV induced acute pancreatitis [29]. In a German study of 166 patients with confirmed PUUV infection, serum lipase levels were elevated in 15% of patients at the acute phase of the disease [30]. Still, abdominal ultrasound or computer tomography (CT) scan showed no signs of acute pancreatitis. Patients with elevated lipase levels did not have abdominal pain more often than those with normal lipase levels, whereas they had a higher serum creatinine concentration, reflecting the severity of AKI [30].

In our previous study, we showed that current smoking is associated with severe AKI in PUUV infection [31]. Several lines of evidence have also indicated a significant association between alcohol consumption and smoking [32,33,34]. Excessive alcohol drinking raises serum liver enzymes, and it increases the risk of acute pancreatitis [35,36]. In addition to smoking, alcohol affects the host’s immune defense. Alcohol use is known to predispose to various infections, such as bacterial pneumonia and systemic bacterial infections [37,38]. In animal models, alcohol exposure also predisposes to viral infections, such as respiratory syncytial virus pneumonia [39]. Furthermore, heavy drinking worsens the clinical outcome of several infections such as pneumonia, tuberculosis, and viral hepatitis [40]. However, there is no data on the possible association between alcohol consumption and the clinical outcome of PUUV infection or other hantavirus infections.

Gamma-glutamyltransferase, GGT, is an enzyme of the liver that has a 70–73% sensitivity to detect heavy alcohol consumption [41]. Another commonly used marker of alcohol consumption is carbohydrate-deficient transferrin, CDT, which has high specificity, but relatively low sensitivity [42]. Using a combination marker of GGT and CDT, formulated with the mathematical equation, the specificity and the sensitivity can be improved [43]. Another biochemical marker of alcohol consumption is ethyl glucuronide (EtG). It is a specific metabolite of ethanol, which can be measured from bodily fluids for several days after cessation of ethanol intake [43].

This study aimed to evaluate whether alcohol drinking affects the clinical outcome of PUUV infection. We also aimed to evaluate the changes in liver and pancreatic enzymes during acute PUUV infection and to assess their associations with patients’ symptoms and other clinical and laboratory findings.

## 2. Materials and Methods

### 2.1. Subjects and Study Protocol

All patients were treated at Tampere University Hospital between January 2005 and November 2014 with serologically confirmed NE. The study cohort consisted originally of 86 consecutive patients of which 66 had control-phase serum samples available, and they were finally included in the study. All patients gave informed consent to participate, and the study protocol was approved by the Ethics Committee of Tampere University Hospital (R04180, R09206, R11188). The samples were collected daily during hospital care from day one at the hospital until discharge or until day five, whichever came first. Clinical variables were obtained during the hospital stay and symptoms were recorded accurately. Convalescent phase samples of patients were obtained on median 24 (range 17–76) days after the onset of fever.

### 2.2. Laboratory Markers and Clinical Variables

Acute PUUV infection was serologically confirmed in all patients [44]. Blood leukocyte and platelet count, hematocrit, and plasma CRP concentration were determined by standard clinical chemical methods at the Laboratory Centre of Pirkanmaa Hospital District (later named Fimlab Laboratories), Tampere, Finland. The concentrations of serum alanine aminotransferase (S-ALT), serum gamma-glutamyl transferase (S-GGT), serum and urine (U) creatinine (S/U-creatinine), serum cystatin C (S-Cys-C), serum carbohydrate-deficient transferrin (S-CDT), serum and urine amylase (S/U-AMYL), urine trypsinogen (U-Trypsin), and urine ethyl glucuronide (U-EtG) were measured by accredited methods at the Laboratory of Seinäjoki Central Hospital, Seinäjoki, Finland. To detect recent alcohol consumption before sampling, U-EtG values above a cut-off limit of 500 ng/mL were used [45]. To improve sensitivity and specificity to detect heavy alcohol drinking, a combination marker of GGT and CDT was used; and, GGT-CDT was counted using a mathematical equation of GGT-CDT = 0.8 × ln(GGT) + 1.3 × ln(CDT). The cut-off limits of 4.3 for men and 3.8 for women were used [46]. Convalescent phase values of GGT-CDT were used to avoid the confounder effect of acute infection which may raise liver enzymes. Both alcohol markers (GGT-CDT and U-EtG) were available from 66 patients at the convalescent phase. Original data is available as Supplementary Material.

The highest or the lowest values of the variables measured during the hospital stay were designated as the maximum or minimum values. The severity of PUUV infection was evaluated with several variables. Cystatin C concentrations and the maximum serum creatinine reflected the severity of AKI. The severity of overall infection was also evaluated with a maximum blood leukocyte count and a minimum platelet count. Weight change during the hospital stay reflected fluid retention and, thus, both a change in the capillary permeability and the severity of AKI, whereas the length of hospital stay reflected the overall severity of the illness. Severe AKI was defined by a creatinine level equal to or more than 353.6 μmol/L which meets the criteria of KDIGO Clinical Practice Guidelines for AKI Stage-3 [47].

The definition of acute pancreatitis consisted of the following criteria: upper abdominal pain and serum amylase elevation threefold above the upper limit of normal [35]. Urine amylase/creatinine and trypsin/creatinine ratios were calculated to eliminate the bias due to urine concentration alterations. The detection limit for U-trypsin was 1.56 ng/mL, and concentrations below the detection limit were regarded as 1.56 ng/mL in the analyses. Clinical variables such as weight, blood pressure, and length of hospital stay, as well as symptoms, were documented accurately during the hospital stay. Cigarette smokers were classified into current smokers and non-smokers (including never smokers and ex-smokers).

### 2.3. Definition of Alcohol Use

We classified patients into three different categories. Patients were classified as alcohol heavy drinkers if the combination marker GGT-CDT exceeded the cut-off limit at the control stage. Patients with a U-EtG level above the cut-off limit but with GGT-CDT below the cut-off limit were classified as light drinkers. Patients who had no elevation in either of these markers were designated as abstainers. Patients were also divided into two groups: alcohol drinkers (including heavy and light drinkers) and abstainers.

### 2.4. Statistical Analysis

Data analyses were performed using IBM SPSS Statistics for Windows, version 27.0 (IBM Corporation, Armonk, NY, USA). To describe the data, medians and ranges were given for skewed continuous variables and numbers and percentages for categorical variables. To compare quantitative variables, a Mann–Whitney U-test was used. Categorical data were analyzed by the Chi-square test or the Fisher exact test, as appropriate. Spearman’s rank correlation test was used to find associations between liver and pancreatic enzyme levels and variables reflecting disease severity. When comparing between two groups (acute and convalescence phase values) a Wilcoxon signed-rank test was used, and when comparing three groups, the Kruskal–Wallis test was used. The *p*-values were adjusted by the Bonferroni correction for multiple tests. A two-sided *p*-value of less than 0.05 was regarded as statistically significant.

## 3. Results

The main laboratory findings and clinical characteristics are presented in Table 1. The median age was 41 years (range 22–74) and 36 (55%) of the patients were males. One or more of the following comorbidities was found prior to the onset of the disease in 17 patients: hypertension (*n* = 7), asthma (*n* = 2), rheumatoid arthritis (*n* = 2), type 2 diabetes (*n* = 3), coronary artery disease (*n* = 2), atrial fibrillation (*n* = 2), celiac disease (*n* = 1), transient ischemic attack (*n* = 1), and gastritis (*n* = 1). None of the patients had chronic kidney disease. Thirty-six (55%) patients were current smokers at the time of the onset of the illness. The patients were admitted to the hospital at the median of four days after the onset of fever. The clinical picture was typical of PUUV infection in all patients.

Out of 66 patients, 43 (65%) had at least one gastrointestinal (GI) symptom at the time of admission to the hospital. Thirty-eight patients (58%) had nausea, 33 (50%) vomited, and 18 (27%) had abdominal pain. When considering the markers reflecting alcohol consumption, S-GGT was found to be over the cut-off limit in 35/66 (53%) of the patients and S-CDT in 2 (3%) patients at the acute stage of NE. Both GGT and CDT were significantly higher during the acute phase when compared with the convalescence phase as shown in Table 2. We also calculated the magnitude of change between acute and control stages of the values presented in Table 2, and we compared these between alcohol drinkers and abstainers. This comparison was also made between heavy drinkers, light drinkers, and abstainers. There were no differences between the two groups (data not shown).

To improve the sensitivity and the specificity to identify heavy drinking, we calculated the combination marker GGT-CDT. In the analyses, we used GGT-CDT of 66 patients at the convalescent phase to avoid the confounder effect of acute infection, which may raise S-GGT. This combination marker showed elevated levels in 5/36 (14%) males and 5/30 (17%) females. Only one patient had high urine EtG (2786 ng/mL) already at admission; while, at the convalescent phase, 20 out of 66 (30%) patients showed urine EtG concentration >500 ng/mL, suggesting recent alcohol consumption. Using either a U-EtG (showing recent alcohol consumption) or a GGT-CDT-combination marker (showing heavy alcohol use) exceeding the cut-off limit, we found 27/66 (41%) patients with biochemical evidence of alcohol consumption before the control visit: 15/36 (42%) males and 12/30 (40%) females.

The median of maximum serum creatinine and cystatin C concentration at the acute phase did not differ between alcohol drinkers as compared with abstainers: S-creatinine 118 µmol/L (range 53–1148) vs. 200 µmol/L (range 62–1139), *p* = 0.208 and S-cystatin C 1.7 mg/L (range 0.8–5.0) vs. 1.9 mg/L (range 0.8–6.5), *p* = 0.270, respectively. There were no differences in other variables reflecting disease severity or gastrointestinal symptoms at admission (data not shown). Alcohol drinkers had slightly higher GGT as compared with abstainers: 57 U/L (range 18–295) vs. 50 U/L (range 16–549), *p* = 0.043. Fifty-two percent (14/27) of alcohol drinkers were current smokers, whereas 56% (22/39) of patients with no alcohol use smoked (*p* = 0.715).

We also compared heavy alcohol drinkers, light drinkers, and abstainers. There were no differences in a length of hospital stay, change in body weight, blood pressure on admission, the lowest platelet count, or in the severity of AKI between these groups as shown in Table 3.

Severe AKI (creatinine >353.6 μmol/L) was not more frequent among heavy drinkers (2/10, 20%) or light drinkers (4/17, 24%) as compared with abstainers (13/39, 33%), *p* = 0.607. Clinical symptoms did not differ either between heavy alcohol drinkers, light drinkers, or abstainers (Table 3). We also wanted to compare heavy alcohol drinkers to the rest of the patients (light drinkers and abstainers). There were no differences in variables reflecting the disease severity, such as the length of hospital stay or the minimum platelet count. In the same way as with other comparisons, neither did the severity of AKI nor clinical symptoms differ between heavy users and other users (data not shown).

Serum ALT exceeded the upper normal limit in 24/66 (36%) and S-GGT in 35/66 (53%) patients at the acute phase of the disease. Both enzymes were elevated in 17/66 (26%) of the patients and one or the other in 42/66 (64%) of the patients. As shown in Table 2, the maximum median ALT and GGT concentrations were significantly higher during the acute phase compared with the values at the control visit. Neither ALT nor GGT correlated with the severity of AKI, lowest platelet count, or any other variable reflecting the severity of the acute infection (data not shown).

The serum amylase concentration was below the normal limit (120 U/L) in almost all patients (62/66, 94%) at the acute phase. None of the patients had major increases in the amylase levels and, interestingly, serum amylase levels were found to be significantly lower during the acute phase when compared with the convalescent phase (Table 2). The highest trypsinogen value in this cohort being 33.7 ng/mL, U-Trypsin was not elevated in any of the patients. All patients had U-trypsin/creatinine ratios within the normal limits.

Patients presenting with vomiting had higher S-ALT than those who did not: 45 U/L (range 17–400) vs. 31 U/L (range 14–87), *p* = 0.009. Otherwise, S-ALT or S-GGT levels did not differ between patients with or without GI symptoms. Patients with abdominal pain on admission showed higher serum amylase activities: 66 U/L (range 33–134) vs. 40 U/L (range 13–181), *p* = 0.006; but, no one showed serum amylase elevation threefold the upper limit of normal. They also showed higher serum creatinine and cystatin C levels compared with those without abdominal pain: 350 µmol/L (range 76–1139) vs. 133 µmol/L (range 53–1148), *p* = 0.020 and 2.1 mg/L (1.2–6.5) vs. 1.6 mg/L (0.8–5.4), *p* = 0.087, respectively.

## 4. Discussion

In the present study, forty-one percent of the patients showed some biochemical signs of alcohol use at the control visit. However, alcohol drinking was not found to show a significant relationship with the clinical course of PUUV infection. Liver enzymes were also commonly elevated in these patients, but they did not associate with the clinical outcome or kidney dysfunction. There were no cases of acute pancreatitis, and serum amylase levels were actually lower in the acute phase of the infection when compared with the convalescent stage.

Patients with evidence of alcohol consumption did not have a more severe clinical course of the acute infection. An enzyme of the liver, GGT is known to reflect alcohol overconsumption with a sensitivity of 70–73% [41]. However, GGT may also rise due to other factors including obesity, smoking, drug use, or some infections, which hampers its use as an alcohol marker, especially in hospital settings. Another widely used marker of alcohol abuse is CDT, which predicts heavy consumption with relatively high specificity but low sensitivity [42]. Both S-GGT and S-CDT are markers of chronic alcohol abuse, and they normalize within two to three weeks after the cessation of alcohol consumption [42,43].

A combination marker of GGT and CDT is more sensitive and specific to detect heavy alcohol consumption than either of these markers alone [42]. The combination marker GGT-CDT reacts when regular ethanol consumption exceeds 40 g per day [43]. In this work, we used the convalescence phase GGT-CDT levels for assessing alcohol consumption because we wanted to exclude the possible confounding effect of GGT elevation due to acute infection. This combined marker indicated heavy alcohol drinking in 14% of males and 17% of females. According to a large Finnish survey, with 13 976 individuals in the national FINRISK-study, the prevalence of heavy alcohol drinking in the general population was 4.2% [48]. In that study, alcohol consumption was assessed with a questionnaire, which may underestimate the true consumption. Nevertheless, our study showed over three times more cases with biomarker-based evidence of heavy alcohol consumption among the present patient population.

Some previous studies have shown that alcohol worsens the clinical outcome in infectious diseases [40]. In this cohort, neither heavy nor light alcohol use was found to predispose to more severe PUUV infection. The severity of AKI did not differ in alcohol drinkers compared with abstainers and there was no difference in other variables reflecting disease severity either. Alcohol drinkers did not show more frequent abdominal pain, vomiting, or nausea on admission to the hospital.

Ethyl glucuronide (EtG) is a specific metabolite of ethanol, which can be measured from bodily fluids for several days after cessation of ethanol intake [45]. Urine EtG can rise after a single instance of use, and it does not necessarily point to chronic alcohol abuse. With the cut-off limit of 500 ng/mL, other sources of ethanol exposure, such as the use of alcohol-containing hand disinfection or alcohol-containing cosmetics, are excluded. In the present study, urine EtG was elevated only in one patient at the acute phase of NE, whereas at the recovery stage 30% of the patients showed a urine EtG concentration over 500 ng/mL. Patients were admitted to the hospital at the median of four days after the onset of the fever. Since the half-life of EtG is short, the acute-phase levels reflect only the consumption of alcohol during the febrile stage of NE. It seems obvious that at the acute phase most patients did not use alcohol; and, therefore, only one patient showed signs of alcohol consumption for a few days before hospitalization. On the other hand, at the convalescent stage, alcohol consumption had probably returned to the usual levels of habitual alcohol intake. Using either urine EtG or GGT-CDT-combination marker as indicators of ethanol consumption, we found that forty-one percent of the patients had used alcohol before the control visit. These patients, however, did not differ from abstainers with respect to clinical symptoms or the course of the disease. Cigarette smoking was common, but there was no difference in smoking status between alcohol drinkers and abstainers.

Liver enzymes, either GGT or ALT, were elevated in 64% of the patients. There were only a few patients with liver enzymes exceeding three-fold the upper limit of normal. In clinical practice, it is often seen that liver enzymes rise slightly during the acute course of many infections and then normalize without any specific treatment. Raised aminotransferases are typically reported during the acute phase of PUUV infection, but the clinical relevance of this observation is not clear [18,19,20,21]. While PUUV has been detected in liver from the lymphocytes of the sinusoids [49], PUUV has not been detected from hepatocytes, to our knowledge. Therefore, PUUV may induce inflammation in the liver without infecting the hepatocytes. Libraty and colleagues have previously shown that patients with severe AKI did not have higher serum ALT levels during acute PUUV infection, when compared with those with non-severe AKI [50]. In the present study, there was neither correlation between serum creatinine nor cystatin C levels and the levels of liver enzymes. Serum aminotransferases did not correlate with other markers of disease severity either. While alcohol abuse is also known to raise S-ALT levels [36], in this study the individuals classified as alcohol drinkers did not have high S-ALT activities, indicating that the quantities of alcohol consumed were probably below the thresholds that would cause significant liver damage.

Serum amylase concentrations in the present material were found to be low in almost all patients and, interestingly, the lowest serum amylase activities occurred at the acute phase of the disease. Patients sought treatment at a median of four days after the onset of fever. Presumably, patients had been feeling sick and lost appetite before admission to the hospital, and we speculate that preceding low nutrient intake due to acute illness may have contributed to the lower amount of salivary and total amylase levels in the acute phase.

Acute pancreatitis is associated with many infections, as also supported by recent observations among patients with SARS-CoV-2-infection [51]. The definition of acute pancreatitis consists of two out of three of the following criteria: (1) upper abdominal pain, (2) serum amylase or lipase elevation threefold above the upper limit of normal, and (3) computed tomography/magnetic resonance imaging/ultrasound findings of pancreatitis [35]. In this study, the definition of acute pancreatitis consisted of the following criteria: upper abdominal pain and serum amylase elevation threefold above the upper limit of normal. None of the patients met these criteria. Urine trypsinogen gives 100% specificity for acute pancreatitis using the cut-off limit of 100 ng/mL [52]. The highest trypsinogen value in this cohort reached 33 ng/mL, also ruling out cases of acute pancreatitis. There are reports of Hantaan, Dobrava, and Seoul virus-induced HFRS accompanied with acute pancreatitis [22,23,24,25,26,27,28]. In Europe, one study of 166 PUUV infected patients did not show any cases of pancreatitis [30]. Based on our findings and previous finding of Kitterer and colleagues [30], it seems that HFRS caused by PUUV does not induce acute pancreatitis.

Sixty-five percent of the patients had some gastrointestinal symptoms at the time of admission to the hospital. Abdominal pain is a common symptom in hantavirus infections for which there is no pathophysiological explanation, so far. A German study found the PUUV nucleocapsid antigen in 62% analyzed intestinal biopsies [53] and in the appendix of one patient [54], reflecting a generalized PUUV infection. In our study, patients with abdominal pain on admission had higher serum amylase and creatinine concentrations than patients without such symptoms. Greater amylase in these patients may be caused by impaired renal function as amylase is eliminated through the kidneys.

There are some limitations in this study, which must be acknowledged. We did not include systematic questionnaire-based interviews of the history and current use of alcohol, such as the Alcohol Use Disorders Identification Test (AUDIT). Another limitation is the absence of other biomarkers, such as phosphatidylethanol (PEth). While PEth is currently known as a highly specific and sensitive biomarker of recent alcohol consumption, fresh blood samples are required for the analysis of this metabolite from red cells that were not available for this study [55]. The GGT-CDT combination marker, however, lacks only little in sensitivity (94%) compared with PEth [55]. Future studies should also address the possibility of whether the use of other biomarkers, such as mean corpuscular volume (MCV), could provide additional value in detecting alcohol consumption and its relation to disease severity in PUUV infected patients [56,57]. The serum and urine samples were frozen before the analyses were made. Freezing might affect the concentrations of some enzymes, but the time of freezing did not differ between acute and control phase samples, and we therefore we find the comparison of these values reliable. The study size is relatively small, but it is sufficient to detect a strong correlation. Further studies with a larger population would be of interest.

In conclusion, in the present study population, forty-one percent of the patients showed biomarker-based signs of recent alcohol consumption at the control visit. Alcohol use did not, however, affect the clinical outcome of the infection, and alcohol users did not have a more severe AKI. There were no cases of acute pancreatitis caused by PUUV infection. Unexpectedly, the serum amylase concentration was significantly lower during the acute phase compared with the control visit, which warrants further studies in larger materials.

## Figures and Tables

**Table 1:** Clinical and laboratory characteristics of 66 patients with acute PUUV infection.

Variable	Median	Range
Clinical variable		
Age (years)	41	22–74
BMI (kg/m^2^) (*n* = 57)	26	19–37
Change in body weight (kg)	2	0–11
Length of hospital stay (days)	6	2–16
Systolic blood pressure on admission (mmHg)	126	72–182
Diastolic blood pressure on admission (mmHg)	80	40–110
Laboratory value		
Creatinine max (μmol/L)	175	53–1148
Cystatin C max (mg/L)	1.9	0.8–6.5
CRP max (mg/L)	91	16–267
Haematocrit max	0.44	0.33–0.60
Leukocyte count max (×10^9^/L)	10.5	4.2–45
Platelet count min (×10^9^/L)	53	5–150

Abbreviations: Max, maximum value during hospital stay; Min, minimum value during hospital stay; BMI, body mass index; CRP, C-reactive protein.

**Table 2:** Comparison of acute-phase values and control values. Control samples were taken at the median of 24 (range 17–76) days after the onset of fever.

	Acute Phase (*n* = 66)		Convalescent Phase (*n* = 66)		
	Median	Range	Median	Range	*p* Value ^a^
ALT max (U/L)	35	14–400	27	5–126	0.003
GGT max (U/L)	52	16–549	44	5–168	0.007
Amyl max (U/L)	48	13–181	74	20–215	<0.001
Trypsinogen max (ng/L)	6.6	1.6–33.7	1.6	1.5–11.1	<0.001
Trypsin/crea max	1.1	0.1–14.9	0.2	0.1–3.5	<0.001
CDT max (%)	1.6	1.0–3.4	1.5	1.0–2.8	0.005
GGT-CDT max					
women	3.5	2.7–5.1	3.3	2.7–4.9	0.072
men	3.9	2.5–6.1	3.7	1.8–4.8	0.034

Abbreviations: Max, maximum value during hospital stay; ALT, serum alanine aminotransferase; GGT, serum gamma-glutamyltransferase; Amyl, serum amylase; Trypsin/crea, urine trypsinogen: creatinine ratio; CDT, serum carbohydrate-deficient transferrin; GGT-CDT, combination marker of gamma-glutamyltransferase and carbohydrate-deficient transferrin. ^a^ Comparison between acute and convalescence phase values; Wilcoxon signed-rank test was used.

**Table 3:** Comparison of laboratory values (median of maximum or minimum value) and clinical symptoms among heavy alcohol drinkers, light drinkers, and abstainers.

	Use of Alcohol			
Variable	Heavy Drinker	Light Drinker	Abstainer	*p*-Value ^a^
	**(*n* = 10)**	**(*n* = 17)**	**(*n* = 39)**	
Clinical variable				
Change in body weight (kg)	1.6	3.5	1.8	0.447
Length of hospital stay (days)	5	8	6	0.153
Systolic blood pressure on admission (mmHg)	127	129	124	0.994
Diastolic blood pressure on admission (mmHg)	80	82	86	0.754
Laboratory value				
ALT max (U/L)	33	34	35	0.967
GGT max (U/L)	98	49	50	0.005
Amyl max (U/L)	32	47	49	0.089
Creatinine max (µmol/L)	115	172	200	0.418
Cystatin C max (mg/L)	1.6	1.7	1.9	0.520
CRP max (mg/L)	99	99	82	0.266
Haematocrit max	0.41	0.44	0.45	0.123
Leukocyte count max (×10^9^/L)	11.7	10.7	10.1	0.692
Platelet count min (×10^9^/L)	78	60	43	0.142
Symptom				
Abdominal pain	1/10 (10%)	4/17 (24%)	13/39 (33%)	0.309
Nausea	5/10 (50%)	11/17 (65%)	22/39 (56%)	0.737
Vomiting	4/10 (40%)	9/17 (53%)	20/39 (51%)	0.785

Abbreviations: Max, maximum value during hospital stay; Min, minimum value during hospital stay; ALT, serum alanine aminotransferase; GGT, serum gamma-glutamyltransferase; Amyl, serum amylase; CRP, plasma C-reactive protein; max, maximum value during hospital stay; min, minimum value during hospital stay. ^a^ Comparison between three groups; Kruskal–Wallis test was used. *P*-values have been adjusted by the Bonferroni correction for multiple tests.

## Data Availability

Original data is available as Supplementary Material.

## Supplementary Materials

The following supporting information can be downloaded at: https://www.mdpi.com/article/10.3390/v14030500/s1. Flie S1: Original data.
